# Supplementary material for: Differential effects of follicle-stimulating hormone glycoforms on the transcriptome profile of cultured rat granulosa cells as disclosed by RNA-seq
Source: PLoS One. 2024 Jun 6;19(6):e0293688. doi: 10.1371/journal.pone.0293688 (PMC11156319; doi:10.1371/journal.pone.0293688)
Supplement: S1 Table — (PDF) [file pone.0293688.s001.pdf]

S1 Table. A representative subset of overexpressed genes at 6 hours of FSH glycoform exposure. Rows in *green* color are unique genes for the corresponding glycoform, whereas those in *orange* are genes shared with other glycoforms.

**FSH18/21**			
**Gene Symbol**	**Gene name**	**ENSRNOG**	**LogFC**
Cyp19a1	P450 arom	ENSRNOT00000000212	1.110000000
Lrrc4b	Leucine Rich Repeat Containing 4B	ENSRNOG00000019418	1.233494517
Cthrc1	Collagen triple helix repeat containing 1	ENSRNOG00000004578	1.427198254
Sult1b1	Sulfotransferase Family 1B Member 1	ENSRNOG00000001967	1.880469362
Sbk1	SH3 domain Binding Kinase1	ENSRNOG00000057696	4.074306225

**FSH24**			
**Gene Symbol**	**Gene name**	**ENSRNOG**	**LogFC**
LOC103692716	heat shock protein HSP 90-alpha	ENSRNOG00000007219	1.787932819
LOC100911319	zinc finger protein 36, C3H1 type-like 2-like	ENSRNOG00000050108	2.103606244
AABR07017110.1	Transcription elongation factor A1-like 1	ENSRNOG00000005869	2.403808315
LOC103689931	heterogeneous nuclear ribonucleoprotein A/B	ENSRNOG00000046272	2.895066489
LOC100911515	Triosephosphate isomerase-like	ENSRNOG00000050669	4.586725682

***rec*FSH**			
**Gene Symbol**	**Gene name**	**ENSRNOG**	**LogFC**
Acsbg1	acyl-CoA synthetase bubblegum family member 1	ENSRNOG00000011381	1.141923857
Lhcgr	luteinizing hormone-chriogonadotropin receptor	ENSRNOT00000022481	1.220000000
Ly9	lymphocyte antigen 9	ENSRNOG00000025069	1.453417476
LOC100911319	zinc finger protein 36, C3H1 type-like 2-like	ENSRNOG00000050108	1.806081224
Cpne1	copine 1	ENSRNOG00000050864	3.235120002

***eq*FSH**			
**Gene Symbol**	**Gene name**	**ENSRNOG**	**LogFC**
LOC103692716	heat shock protein HSP 90-alpha	ENSRNOG00000007219	1.756211659
LOC100911319	zinc finger protein 36, C3H1 type-like 2-like	ENSRNOG00000050108	2.149543106
LOC103689931	heterogeneous nuclear ribonucleoprotein A/B	ENSRNOG00000046272	2.818252315
LOC100911515	triosephosphate isomerase-like	ENSRNOG00000050669	4.314492925
LOC100365839	40S ribosomal protein S3a-like	ENSRNOG00000048109	6.882034523
