# Supplementary material for: Differential effects of follicle-stimulating hormone glycoforms on the transcriptome profile of cultured rat granulosa cells as disclosed by RNA-seq
Source: PLoS One. 2024 Jun 6;19(6):e0293688. doi: 10.1371/journal.pone.0293688 (PMC11156319; doi:10.1371/journal.pone.0293688)
Supplement: S2 Table — (PDF) [file pone.0293688.s002.pdf]

S2 Table. A representative subset of overexpressed genes at 12 hours of FSH glycoform exposure. Rows in *green* color are unique genes for the corresponding glycoform, whereas those in *orange* are genes shared with other glycoforms.

**FSH18/21**			
**Gene Symbol**	**Gene name**	**ENSRNOG**	**LogFC**
Nppc	natriuretic peptide C	ENSRNOG00000018854	2.097631300
Acsbg1	Acyl-CoA synthetase bubblegum family member 1	ENSRNOG00000011381	2.199123724
Tmem178b	transmembrane protein 178B	ENSRNOG00000047635	2.539311373
Lhcgr	Luteinizing hormone-chriogonadotropin receptor	ENSRNOT00000022481	3.617129804
Cyp19a1	P450arom	ENSRNOG00000000196	4.196460356

**FSH24**			
**Gene Symbol**	**Gene name**	**ENSRNOG**	**LogFC**
Acsbg1	acyl-CoA synthetase bubblegum family member 1	ENSRNOG00000011381	2.080032062
Tmem178b	transmembrane protein 178B	ENSRNOG00000047635	2.286677054
Lhcgr	Luteinizing hormone/choriogonadotropin receptor	ENSRNOT00000022481	3.203317271
Cyp19a1	P450arom	ENSRNOG00000000196	3.340484357
Inmt	indolethylamine N-methyltransferase	ENSRNOG00000011250	3.730104998

***rec*FSH**			
**Gene Symbol**	**Gene name**	**ENSRN OG**	**LogFC**
Srcin1	SRC kinase signaling inhibitor 1	ENSRNOG00000011475	1.86312316
Tmem178a	Transmembrane protein 178A	ENSRNOG00000007907	1.916738806
Tmem178b	Transmembrane protein 178B	ENSRNOG00000047635	2.196115722
Upk1b	Uroplakin 1B	ENSRNOG00000027380	2.458571633
Lhcgr	Luteinizing hormone-chriogonadotropin receptor	ENSRNOT00000022481	2.510000000

***eq*FSH**			
**Gene Symbol**	**Gene name**	**ENSRNOG**	**LogFC**
Tmem178a	transmembrane protein 178A	ENSRNOG00000007907	1.904365537
Abi3bp	ABI family member 3 binding protein	ENSRNOG00000001627	2.033449035
Srcin1	SRC kinase signaling inhibitor 1	ENSRNOG00000011475	2.073592126
Tmem178b	transmembrane protein 178B	ENSRNOG00000047635	2.303801799
Lhcgr	luteinizing hormone-chriogonadotropin receptor	ENSRNOT00000022481	2.716207033
