# Supplementary material for: Differential effects of follicle-stimulating hormone glycoforms on the transcriptome profile of cultured rat granulosa cells as disclosed by RNA-seq
Source: PLoS One. 2024 Jun 6;19(6):e0293688. doi: 10.1371/journal.pone.0293688 (PMC11156319; doi:10.1371/journal.pone.0293688)
Supplement: S3 Table — (PDF) [file pone.0293688.s003.pdf]

S3 Table. A representative subset of underexpressed genes at 6 hours of FSH glycoform exposure. Rows in *green* color are unique genes for the corresponding glycoform, whereas those in *orange* are genes shared with other glycoforms.

**FSH18/21**			
**Gene Symbol**	**Gene name**	**ENSRNOG**	**LogFC**
Serpinb2	serpin family B member 2	ENSRNOG00000002460	-0.933897522
AABR07063424.1	-	ENSRNOG00000060896	-0.860295243
AABR07015080.2	-	ENSRNOG00000059586	-0.859621907
AABR07015078.1	-	ENSRNOG00000055956	-0.85951943
AABR07015066.1	-	ENSRNOG00000046600	-0.859432827

**FSH24**			
**Gene Symbol**	**Gene name**	**ENSRNOG**	**LogFC**
Eif4g1	eukaryotic translation initiation factor 4 gamma 1-like	ENSRNOG00000049598	-2.6181025
LOC108353446	40S ribosomal protein S2 pseudogene	ENSRNOG00000005116	-1.688086149
LOC100911575	60S acidic ribosomal protein P2-like	ENSRNOG00000037607	-1.325378827
Rplp2	ribosomal protein lateral stalk subunit P2	ENSRNOG00000002116	-1.324964798
Rps19	ribosomal protein S19-like2	ENSRNOG00000037897	-1.221079272

**recFSH**			
**Gene Symbol**	**Gene name**	**ENSRNOG**	**LogFC**
Rbm12	RNA binding motif protein 12	ENSRNOG00000046990	-1.967150559
AABR07042077.1	-	ENSRNOG00000002734	-1.160515769
Tox3	TOX high mobility group box family member 3	ENSRNOG00000028649	-1.034259027
Vom2r4	vomeronasal 2 receptor, 4	ENSRNOG00000047388	-0.886585355
Fam131b	family with sequence similarity 131, member B	ENSRNOG00000017149	-0.817911791

***eq*FSH**			
**Gene Symbol**	**Gene name**	**ENSRNOG**	**LogFC**
AABR07002564.1	-	ENSRNOG00000022227	-2.26932217
Lurap1l	leucine rich adaptor protein 1-like	ENSRNOG00000033740	-2.244014822
LOC108353446	40S ribosomal protein S2 pseudogene	ENSRNOG00000005116	-1.543680393
AABR07016640.1	-	ENSRNOG00000037522	-1.497071649
Rplp2	ribosomal protein lateral stalk subunit P2	ENSRNOG00000002116	-1.412925958
