# Supplementary material for: Differential effects of follicle-stimulating hormone glycoforms on the transcriptome profile of cultured rat granulosa cells as disclosed by RNA-seq
Source: PLoS One. 2024 Jun 6;19(6):e0293688. doi: 10.1371/journal.pone.0293688 (PMC11156319; doi:10.1371/journal.pone.0293688)
Supplement: S4 Table — (PDF) [file pone.0293688.s004.pdf]

S4 Table. A representative subset of underexpressed genes at 12 hours of FSH glycoform exposure. Rows in *green* color are unique genes for the corresponding glycoform, whereas those in *orange* are genes shared with other glycoforms.

**FSH18/21**			
**Gene Symbol**	**Gene name**	**ENSRNOG**	**LogFC**
Slc7a11	solute carrier family 7 member 11	ENSRNOG00000010210	-1.847219506
Fosl1	FOS like 1, AP-1 transcription factor subunit	ENSRNOG00000020552	-1.533987983
Hbb-bs	hemoglobin, beta adult s chain	ENSRNOG00000061299	-1.410551353
Plxna2	plexin A2	ENSRNOG00000007324	-1.389753984
Nav3	neuron navigator 3	ENSRNOG00000052157	-1.201679526

**FSH24**			
**Gene Symbol**	**Gene name**	**ENSRNOG**	**LogFC**
Plxna2	plexin A2	ENSRNOG00000007324	-1.425020385
Sema3e	semaphorin 3E	ENSRNOG00000006631	-1.055925692
Niban1	niban apoptosis regulator 1	ENSRNOG00000002403	-1.0311566
Angptl4	Angiopoietin-like 4	ENSRNOG00000007545	-0.968333237
Pvr	PVR cell adhesion molecule	ENSRNOG00000019202	-0.96014559

recFSH			
Gene Symbol	Gene name	ENSRNOG	LogFC
Fosl1	FOS like 1, AP-1 transcription factor subunit	ENSRNOG00000020552	-1.520469579
Plxna2	plexin A2	ENSRNOG00000007324	-1.172556625
Cited1	Cbp/p300-interacting transactivator with Glu/Asp-rich carboxy-terminal domain 1	ENSRNOG00000003189	-1.076087521
Nefm	neurofilament medium	ENSRNOG00000013916	-1.067886743
Dmxl2	Dmx-like 2	ENSRNOG00000009170	-0.918660843

***eq*FSH**			
**Gene Symbol**	**Gene name**	**ENSRNOG**	**LogFC**
Hbb-bs	hemoglobin, beta adult s chain	ENSRNOG00000061299	-1.469645995
Hbb	hemoglobin subunit beta	ENSRNOG00000058105	-1.418580545
St6galnac3	ST6 N-acetylgalactosaminide alpha-2,6-sialyltransferase 3	ENSRNOG00000056894	-1.357981341
Plxna2	plexin A2	ENSRNOG00000007324	-1.301302127
Angptl4	Angiopoietin-like 4	ENSRNOG00000007545	-1.100253745
