# Supplementary figures and images for: Differential effects of follicle-stimulating hormone glycoforms on the transcriptome profile of cultured rat granulosa cells as disclosed by RNA-seq
Source: PLoS One. 2024 Jun 6;19(6):e0293688. doi: 10.1371/journal.pone.0293688 (PMC11156319; doi:10.1371/journal.pone.0293688)

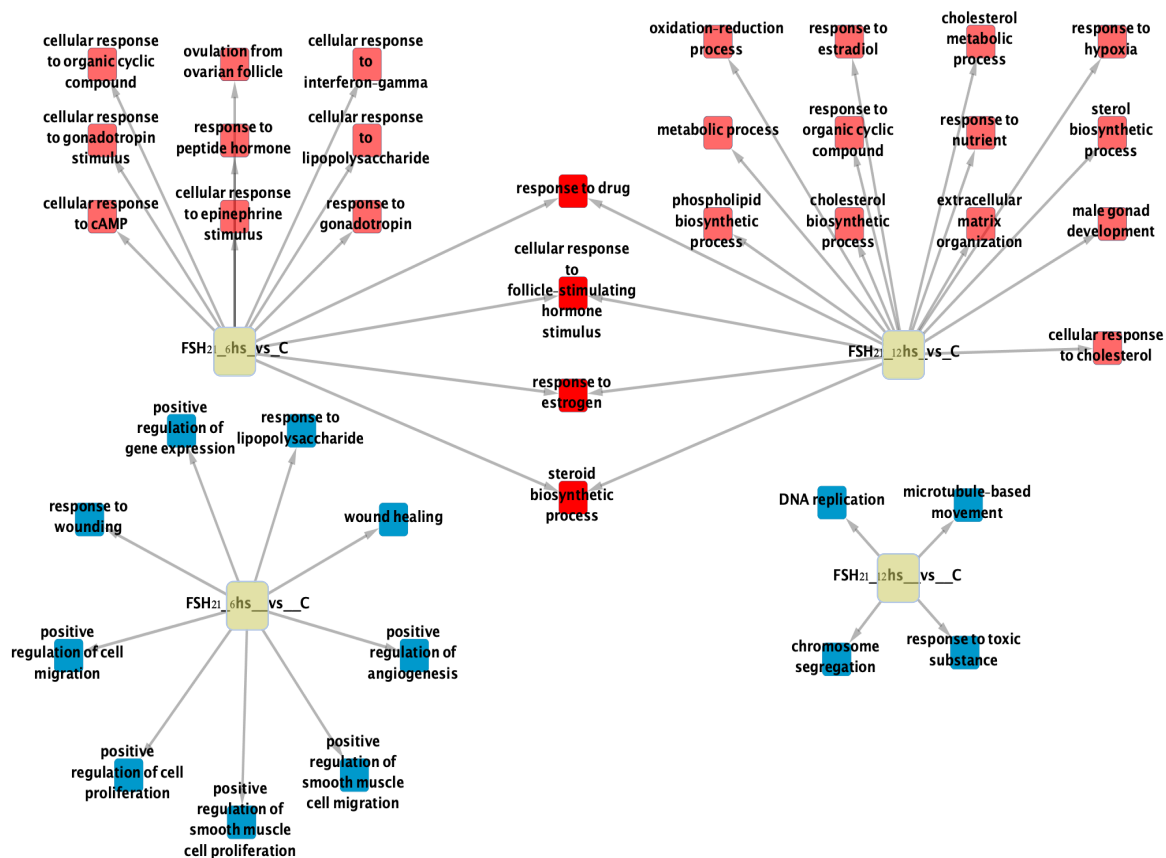

**S1 Fig.**

Supplement: S1 Fig — In this representation, the red color corresponds to overexpressed enriched processes and the blue color to underexpressed processes. The intersection of the overexpressed processes at 6 h is against the underexpressed processes at 12 h, that is, the comparasion is performed between the two incubation times. This is why the exclusive processes may be different than those shown in Fig 4. (PDF) [file pone.0293688.s005.pdf]

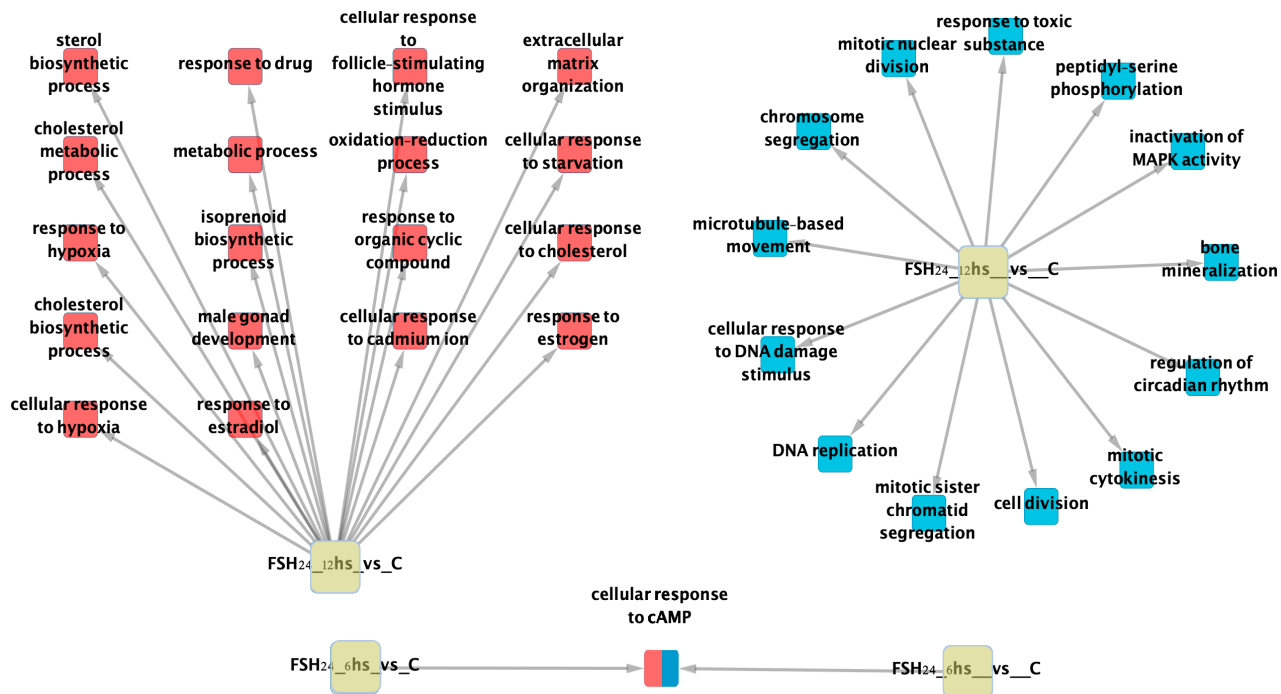

S2 Fig.

Supplement: S2 Fig — In this representation, the red colored squares corresponds to overexpressed enriched processes and the blue color to underexpressed processes. (PDF) [file pone.0293688.s006.pdf]

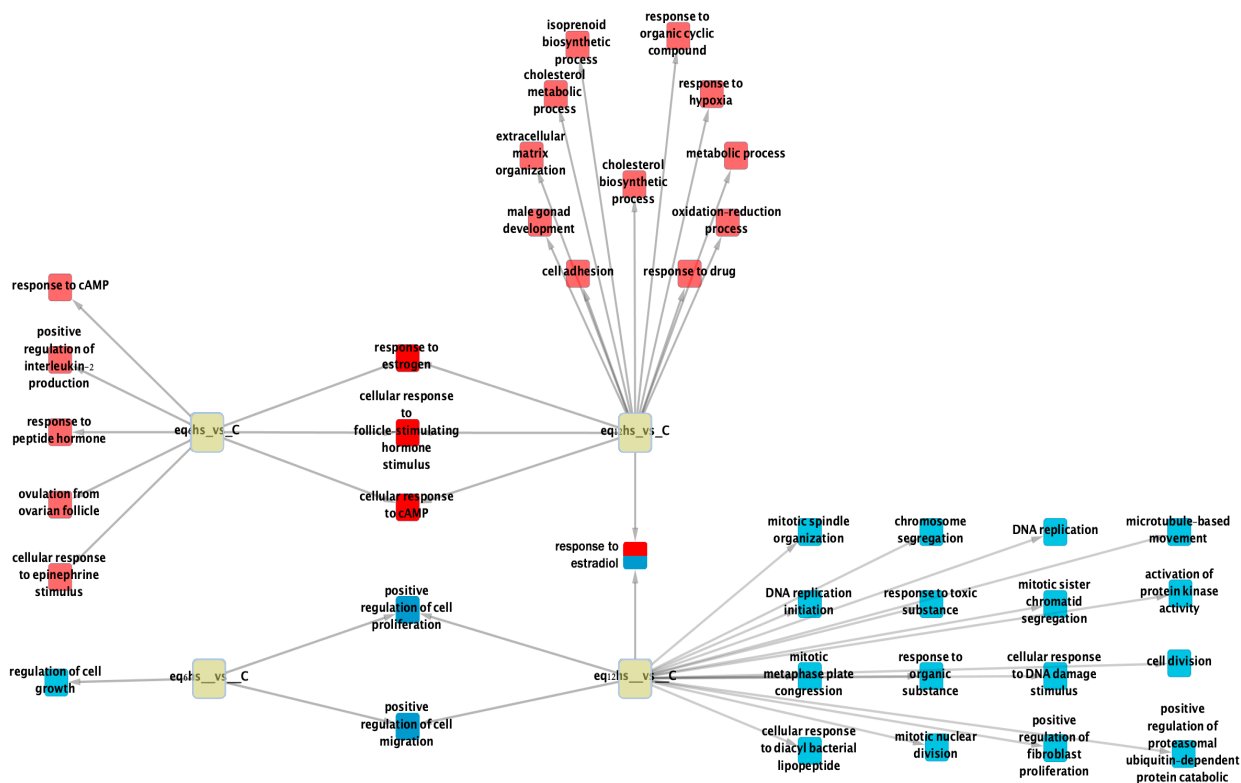

Supplement: S3 Fig — The red squares correspond to overexpressed enriched processes and the blue color to underexpressed processes. (PDF) [file pone.0293688.s007.pdf]

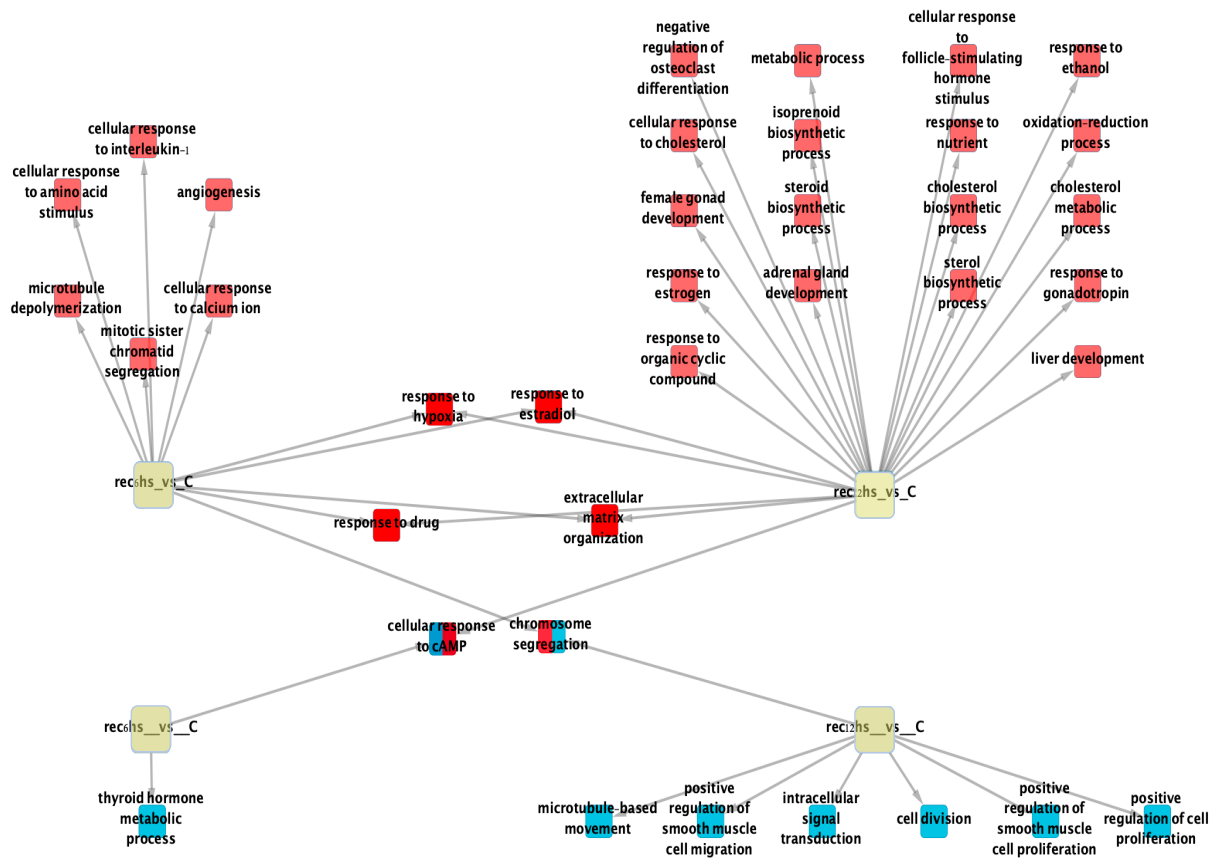

**S4 Fig.**

Supplement: S4 Fig — The red squares correspond to overexpressed enriched processes and the blue color to underexpressed processes. (PDF) [file pone.0293688.s008.pdf]

# cAMP pathway 6h

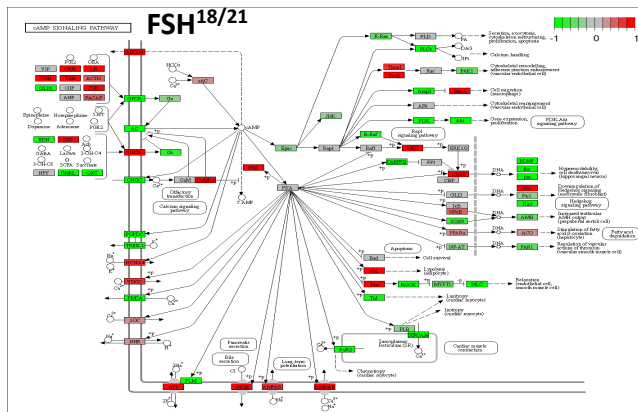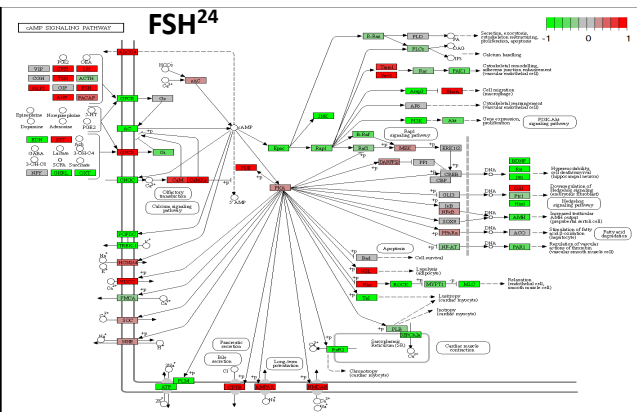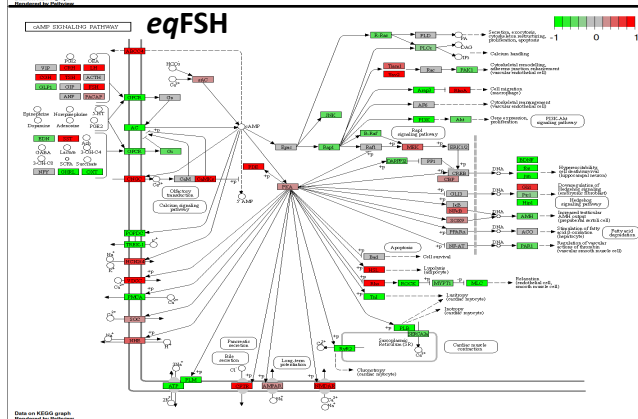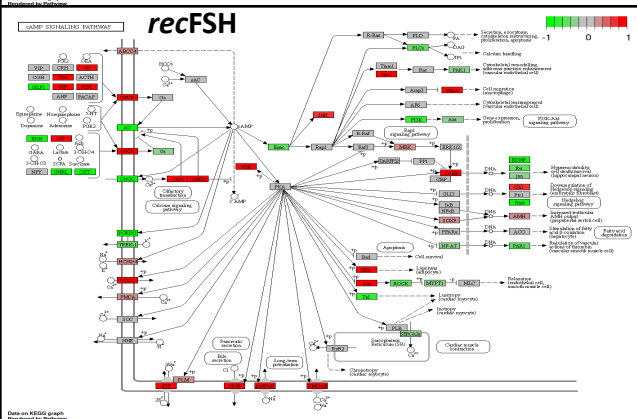

Supplement: S5 Fig — This pathway is significantly perturbed by both FSH18/21 and FSH24 at 6h. Note the activation of CREB followed by an inhibition of c-fos induced by FSH18/21, not observed with FSH24. In eqFSH and recFSH, this pathway is significantly perturbed at 6 h. Note the difference in phosphodiesterase (PDE) states: induced by eFSH, and repressed by recFSH. [Pathway perturbation detected with GAGE. Pathway visualization rendered with Pathview]. (PDF) [file pone.0293688.s009.pdf]

# cAMP pathway 12 h

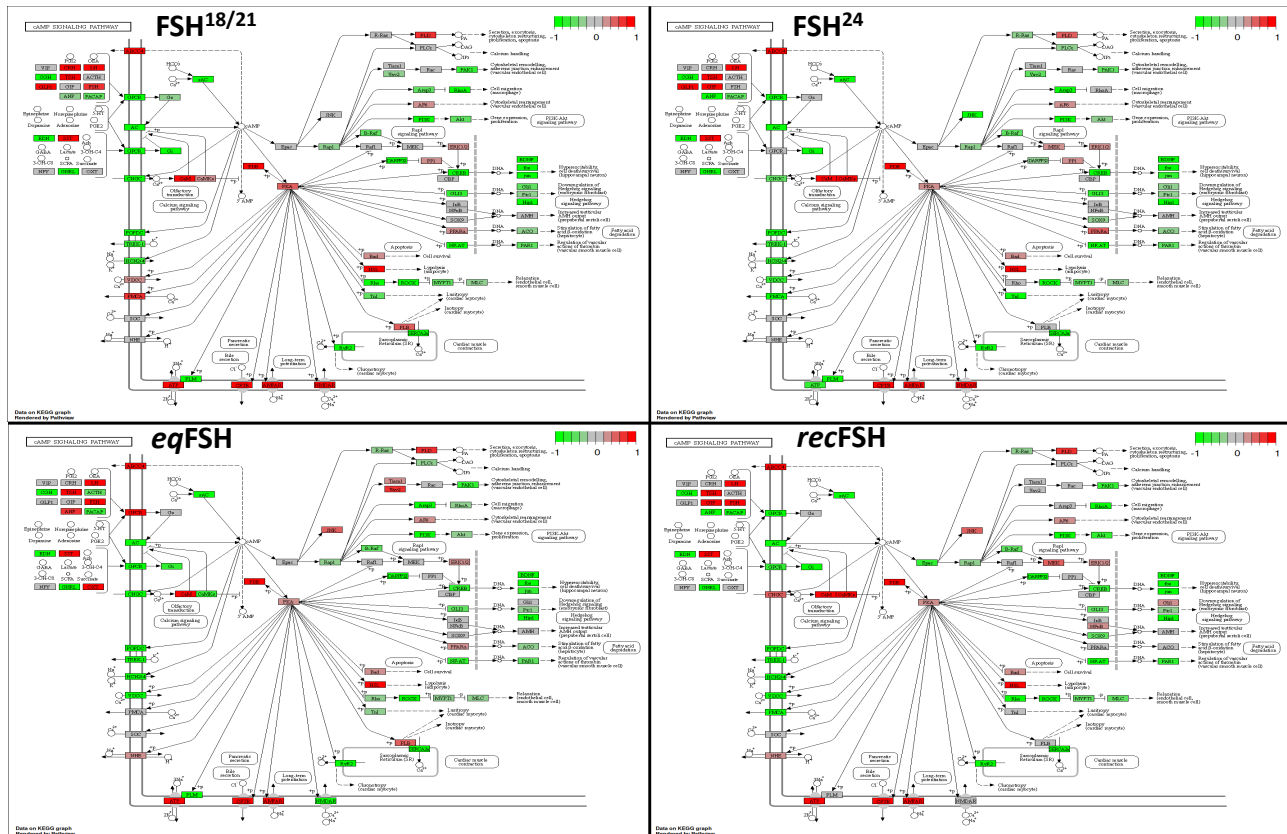

Supplement: S6 Fig — At this time, this pathway was significantly perturbed by both FSH18/21 and FSH24. Note the activation of PAR1 (coagulation factor II thrombin receptor) by FSH24, which was not induced by FSH18/21. This pathway was significantly perturbed by both recFSHand eFSH at this time. In addition, note the activation of adenylate cyclase (AC) and lipase E (HSL) by recFSH, not induced by eFSH. [Pathway perturbation detected with GAGE. Pathway visualization rendered with Pathview]. (PDF) [file pone.0293688.s010.pdf]

# MAPK pathway 6h

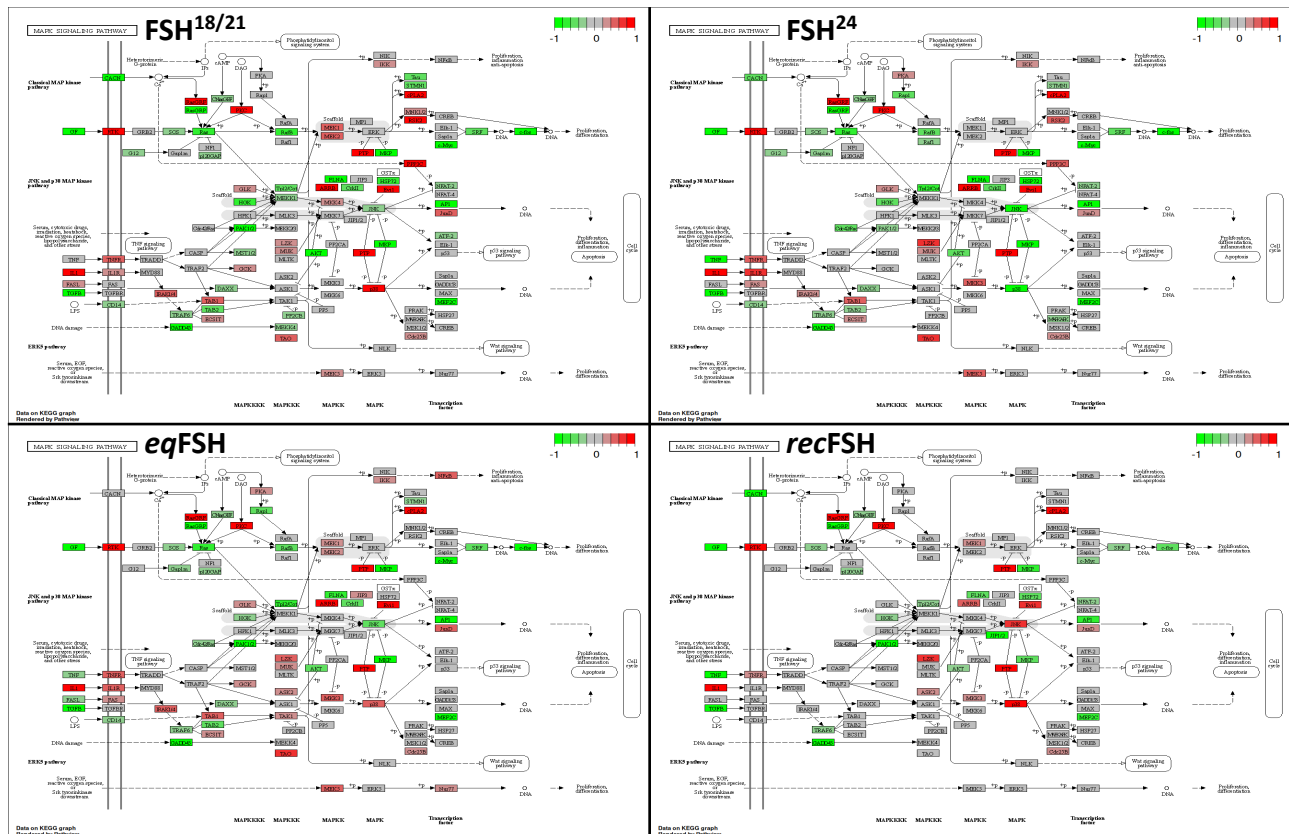

Supplement: S7 Fig — Notably, receptor signaling proteins presented varying expression levels: FSH24 induced a more profound overexpression than FSH18/21 and eqFSH, while recFSH elicited comparatively lower expression. Furthermore, our analysis revealed that highly central molecules within the MAPK pathway, such as p38, responded differentially to the FSH glycoforms. Under the influence of FSH24, p38 and similar central molecules exhibited underexpression. In contrast, when exposed to all other FSH glycoforms, these central molecules (mainly p38) exhibited a consistent pattern of overexpression. [Pathway perturbation was detected using GAGE, and the pathway visualization was generated through Pathview]. (PDF) [file pone.0293688.s011.pdf]

# MAPK pathway 12h

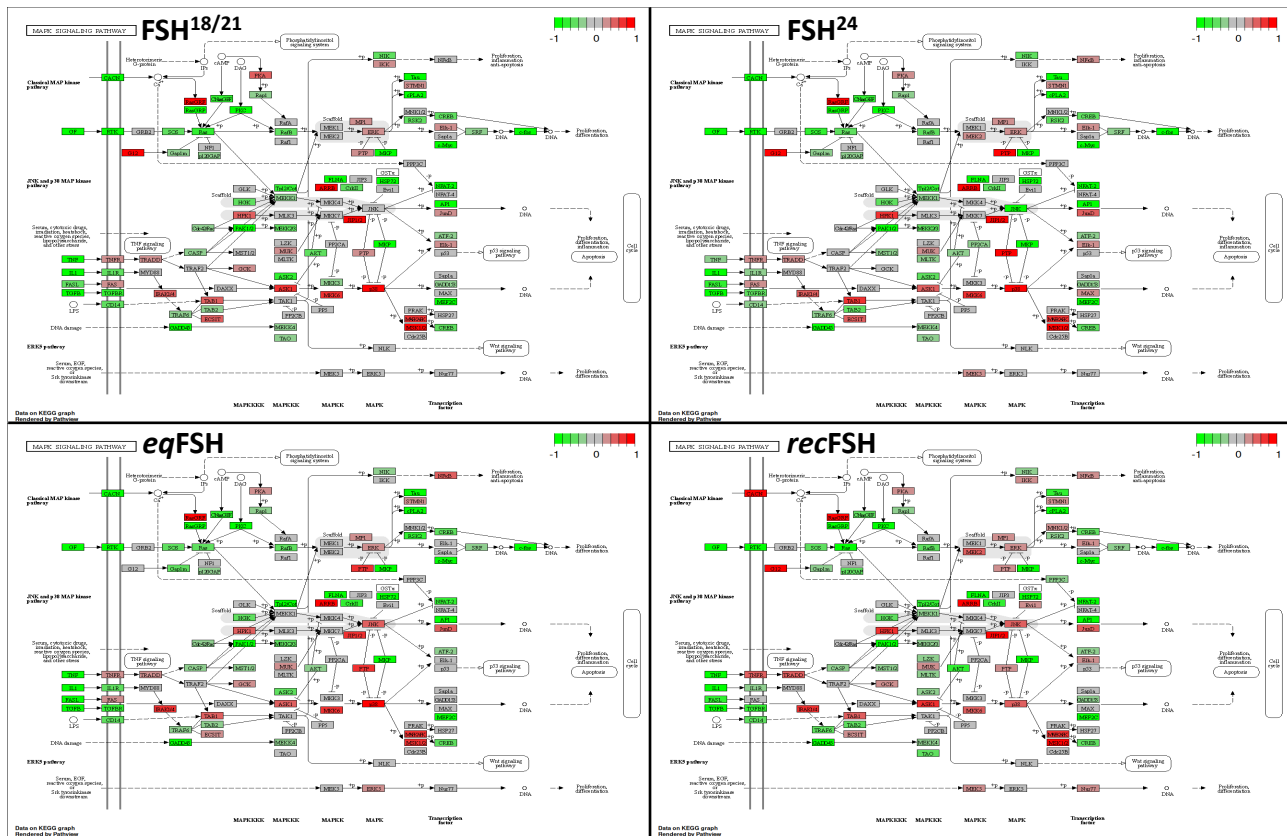

Supplement: S8 Fig — Receptor signaling proteins are now predominantly underexpressed, regardless of the particular FSH treatment applied, with a marked shift from the earlier (6 h) overexpression. Notably, at this time central molecules, like p38, consistently exhibited overexpression across all FSH glycoforms perturbations. [Pathway perturbation was detected using GAGE, and the pathway visualization was generated through Pathview]. (PDF) [file pone.0293688.s012.pdf]

## PI3K-AKT pathway 12h

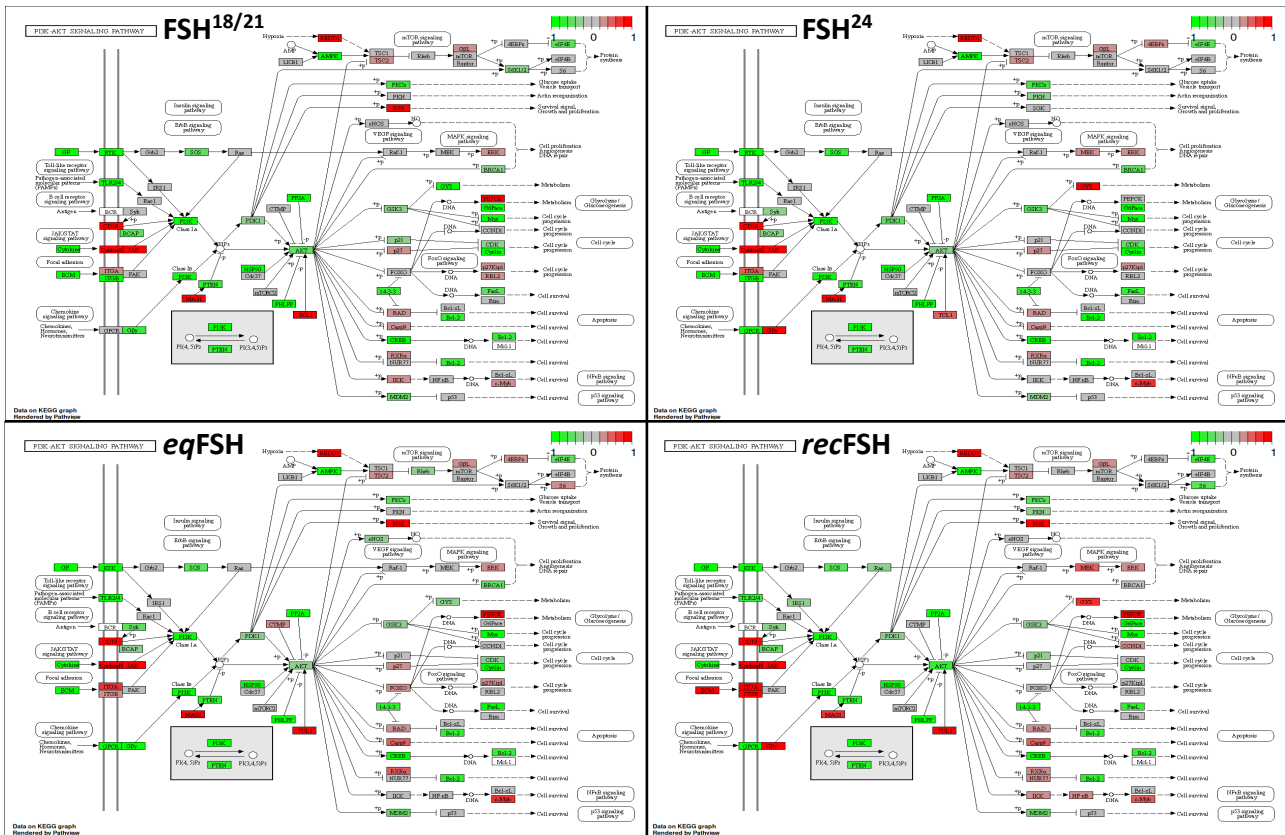

Supplement: S10 Fig — First, expression of signaling molecules exhibited a more varied pattern which was distinctly influenced by each FSH glycoform, suggesting differential regulatory effects. For instance, in the case of the proteins involved in focal adhesion, ECM, ITGA, and ITGB were, respectively: a) all upregulated by recFSH; b) up-, down-, and down-regulated, respectively, by eqFSH; and c) down-, up-, and down-regulated, respectively by FSH18/21 and FSH24. In the case of downstream effectors, the patterns of expression of molecules such as GYS and PEPCK were unique for each glycoform: a) both upregulated by recFSH; b) down- and upregulated, respectively, by eqFSH; c) down- and up-regulated, respectively, by FSH18/21,; and c) up-regulated and without change, respectively, by FSH24. All glycoforms consistently showed underexpression of PI3K persisted across all compounds, indicating a sustained (and shared) impact on this key element. [Pathway perturbation was detected using GAGE, and the pathway visualization was generated through Pathview]. (PDF) [file pone.0293688.s014.pdf]
