# Supplementary material for: Differential effects of follicle-stimulating hormone glycoforms on the transcriptome profile of cultured rat granulosa cells as disclosed by RNA-seq
Source: PLoS One. 2024 Jun 6;19(6):e0293688. doi: 10.1371/journal.pone.0293688 (PMC11156319; doi:10.1371/journal.pone.0293688)
Supplement: S9 Fig — Receptor signaling proteins expression showed a balanced distribution of both up- and down regulation across all FSH glycoforms, with no significant differences. PI3K consistently exhibited underexpression across all glycoforms, indicating a shared regulation. Notably, downstream effectors within the PI3K pathway showed unique responses; recFSH upgregulated effectors such as CCND1, CDK, and cyclin; and eqFSH, FSH18/21 and FSH24 downregulated BCl-2 and c-Myb. [Pathway perturbation was detected using GAGE, and the pathway visualization was generated through Pathview]. (PDF) [file pone.0293688.s013.pdf]

PI3K-AKT pathway 6h

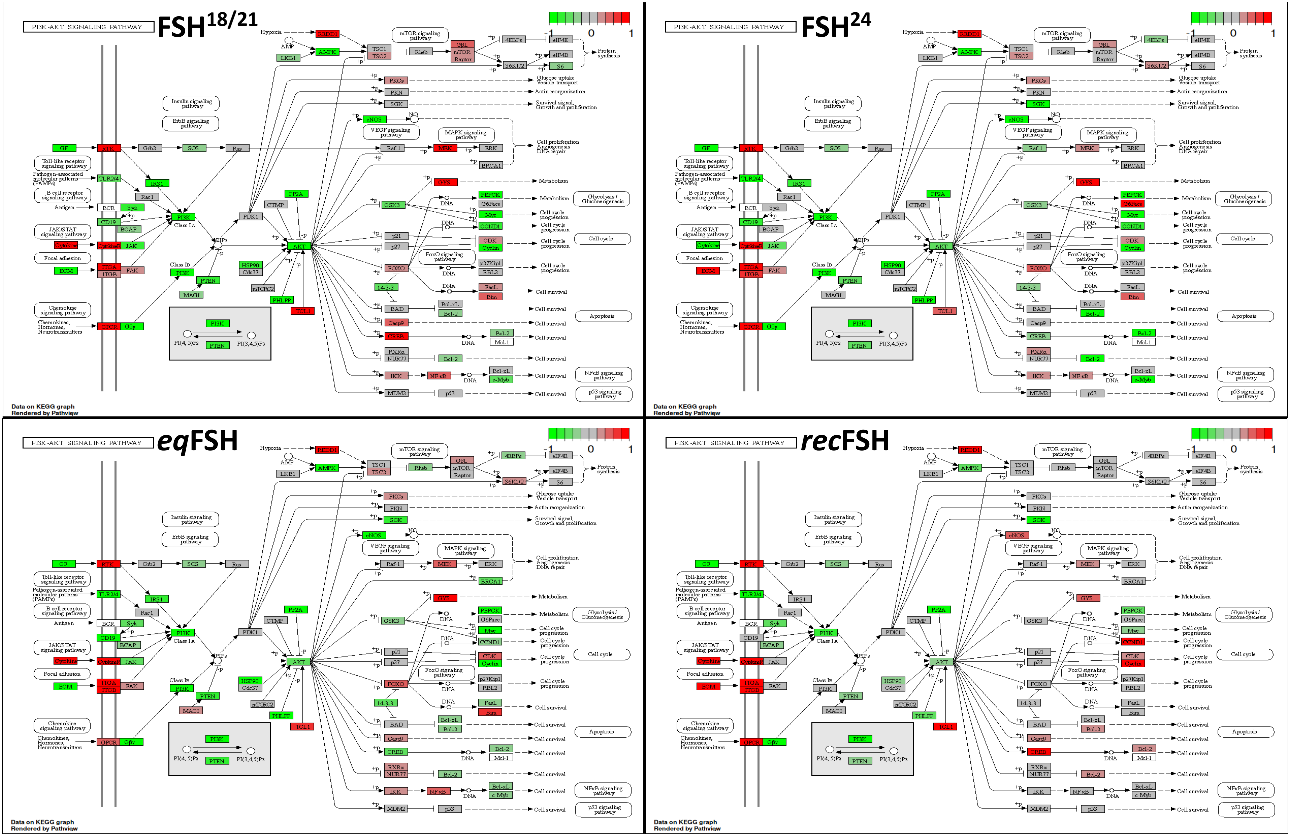

S9 Fig.
